# Laparoscopic skills training: the effects of viewing mode (2D vs. 3D) on skill acquisition and transfer

**DOI:** 10.1007/s00464-020-07923-8

**Published:** 2020-09-02

**Authors:** Kirsty L. Beattie, Andrew Hill, Mark S. Horswill, Philip M. Grove, Andrew R. L. Stevenson

**Affiliations:** 1 School of Psychology, The University of Queensland, St Lucia, Brisbane, 4072 Australia; 2 Clinical Skills Development Service, Metro North Hospital and Health Service, Brisbane, Australia; 3 Minerals Industry Safety and Health Centre, Sustainable Minerals Institute, The University of Queensland, Brisbane, Australia; 4 School of Medicine, The University of Queensland, Brisbane, Australia; 5 Department of Colon and Rectal Surgery, Royal Brisbane and Women’s Hospital, Brisbane, Australia

**Keywords:** 3D laparoscopy, 2D laparoscopy, Surgical skills, Training, Novices, Learning

## Abstract

**Background:**

Three-dimensional (3D) visual displays have been suggested to aid laparoscopic skills training by providing the depth cues not present in traditional two-dimensional (2D) displays. However, few studies have robustly investigated the impact of viewing mode (2D vs. 3D) on learning outcomes.

**Purpose:**

To examine how viewing mode (2D vs. 3D) impacts the acquisition and transferability of basic laparoscopic skills by comparing performance between transfer and control groups on a complete proficiency-based training program.

**Method:**

A counterbalanced between-subjects design was employed. Each participant was randomly allocated to one of four groups, comprising two transfer groups (trained in one viewing mode and tested in the alternate mode: the *2D → 3D* and *3D → 2D* groups) and two control groups (trained and tested in one viewing mode: the *2D → 2D* and *3D → 3D* groups). Participants completed proficiency-based training in six laparoscopic training tasks. Testing included two further repetitions of all tasks under test conditions. Objective performance measures included the total number of repetitions to reach proficiency, and total performance scores (i.e. time + error penalties across all repetitions) in training and testing.

**Results:**

The groups trained in 3D demonstrated superior training performance (i.e. less time + errors) and took fewer repetitions to reach proficiency than the groups trained in 2D. The groups tested in 3D also demonstrated superior test performance compared to those tested in 2D. However, training mode did not yield significant test differences between the groups tested in 2D (i.e. 2D → 2D vs. 3D → 2D), or between the groups tested in 3D (i.e. 3D → 3D vs. 2D → 3D).

**Conclusion:**

Novices demonstrate superior performance in laparoscopic skills training using a 3D viewing mode compared to 2D. However, this does not necessarily translate to superior performance in subsequent testing or enhanced learning overall. Rather, test performance appears to be dictated by the viewing mode used during testing, not that of prior training.

**Electronic supplementary material:**

The online version of this article (10.1007/s00464-020-07923-8) contains supplementary material, which is available to authorised users.

## Background

Laparoscopy is currently considered the gold standard approach for a range of surgical treatments [1]. While these minimally invasive procedures offer significant advantages in patient recovery, they also require complex surgical skills that are difficult for novices to learn and highly vulnerable to error. Moreover, research indicates that 80% of major laparoscopic complications occur early on in a surgeon’s career (often within the first 100 cases), and can lead to disastrous patient outcomes including permanent injury or fatality [2–4]. Consequently, it is important for contemporary training methods to accelerate the learning curve and provide trainees with a platform to further optimise laparoscopic skills within the training period [5]. It has been suggested that the recent introduction of three-dimensional (3D) visual displays has the potential to accelerate the learning curve in training by overcoming the perceptual constraints associated with traditional two-dimensional (2D) displays (i.e. by increasing depth perception) [6]. However, not all contemporary operating theatres have access to 3D displays to support surgical tasks, and different equipment is often employed across sites (e.g. displays of differing brand, size, and picture quality) [7]. As a result, laparoscopic surgeons must be able to demonstrate surgical proficiency with any and all display types.

Evidence suggests that superior performance during training in simulated laparoscopic tasks (i.e. faster completion times and fewer errors) can be achieved when novices use a 3D viewing mode rather than a 2D display or viewing mode [6–9]. However, several studies identified that the superior skill acquisition associated with training in 3D was not reflected in subsequent performance during testing in 2D [7, 10–12]. Further, those trained in 2D performed at a superior level when subsequently tested in 3D [7, 10–12]. Such results have been interpreted as an indication of ineffective skill transfer from 3D to 2D, and effective or increased skill transfer from 2D to 3D [7, 10–12]. If this interpretation is correct, it may be because 2D training forces trainees to attend to and rely upon secondary depth cues (e.g. shadows, object occlusion, image size, and changes with motion) [13] to overcome the perceptual constraints of 2D displays (e.g. an absence of the convergence and stereopsis that normal vision relies on) [13]. Thus, despite the extra difficulty associated with 2D leading to increased training time, subsequent performance in 3D is enhanced as trainees are able to use both primary and secondary depth cues. Similarly, it could also be argued that those who train in 3D may not learn to exploit secondary depth cues due to the availability of primary depth cues, and subsequently experience greater difficulty performing tasks in 2D when primary depth cues are no longer available.

It is also important to note that some studies have found opposing results regarding skill transfer (i.e. transferring from 3D to 2D appeared to improve performance, while transferring from 2D to 3D appeared to reduce performance [14–17]. However, unlike the research discussed above, these studies employed a defined number of repetitions or a fixed length of time for participant training, rather than the attainment of a criterion or “proficiency” level [14–17]. As a result, the 2D- and 3D-trained groups did not reach an equivalent level of performance during training, and thus any comparisons of subsequent performance in alternate viewing modes should be interpreted with caution [14–17]. Unfortunately, drawing clear conclusions from prior research into 2D/3D skill transfer is ultimately problematic due to the omission of appropriate control groups. Even in studies where participants were trained to a proficiency criterion, it is impossible to determine the extent to which observed performance differences on transfer tasks were due to the viewing mode itself or the additional practice generally received by those trained in 2D. In addition, previous research has typically failed to account for potentially confounding variables, such as participants’ stereoacuity (i.e. the smallest detectable difference in depth that they can perceive using binocular vision) [6, 18, 19], their psychomotor abilities (e.g. manual dexterity), or potential “crosstalk” (i.e. double vision due to the use of suboptimal 3D viewing positions, which results in both eyes viewing a combination of the image intended for one eye and a portion of the image intended for the other eye) [19].

The aim of the present study was to overcome the limitations of prior research and draw robust conclusions regarding the impact of viewing mode on laparoscopic training and performance. Specifically, the current study examines (1) whether using a 3D (versus 2D) viewing mode enhances the acquisition of laparoscopic skills, and (2) to what extent the skills acquired using a 3D viewing mode transfer to 2D, and vice versa. Furthermore, as research has yet to determine whether differences in transfer performance can be attributed to the change in viewing mode itself or to the different lengths of exposure during prior proficiency-based practice, this study also examines (3) whether differences in test performance exist between those trained and tested in alternate viewing modes and those who experience only one viewing mode in training and testing. The answers to these questions carry significant implications for the hospitals and clinics currently training laparoscopic surgeons, and for the trainee surgeons themselves. For instance, if training in 3D is found to enhance skill acquisition, then training centres may wish to invest in the latest 3D technology to accelerate the learning curve for trainees. However, if the skills acquired under 3D conditions do not adequately transfer to subsequent performance in 2D, then this could be disadvantageous for trainees who learn in 3D but subsequently work at sites with different (i.e. 2D-only) equipment.

## Materials and methods

### Participants

Sixty novices (male, *n* = 32; female, *n* = 28) with a mean age of 24.78 (*SD* = 3.24, range = 19–34) voluntarily participated in the study. Participants were current medical students (between first and fourth year of medical school), recruited via an advertisement placed on the University of Queensland’s surgical interest group (Incision UQ) noticeboard. All participants reported normal or corrected-to-normal vision, normal stereoacuity, and no prior laparoscopic experience (including no formalised laparoscopic skills training with a simulator, and no hands-on laparoscopic experience in an operational context, e.g. as a surgical assistant). Fifty-six participants were right-handed and four were left-handed. Participants received no compensation for their involvement. The study was approved by the relevant institutional review boards (i.e. the Human Research Ethics Committees of the Royal Brisbane and Women’s Hospital (RBWH) and The University of Queensland).

### Design

A counterbalanced between-subjects design was employed. Each participant was randomly allocated to one of four experimental groups (in blocks of four participants). This comprised two transfer groups (*trained* in one viewing mode → *tested* in the alternate mode: the *2D → 3D* and *3D → 2D* groups) and two control groups (*trained* → *tested* in one viewing mode: the *2D → 2D* and *3D → 3D* groups). Given that some studies have found significant performance differences between sexes during initial laparoscopic skills training [15, 20, 21], a conservative strategy of randomising separately for males and females was used to avoid significantly uneven sex distributions across the groups.

### Procedure

**Fig. 1:**
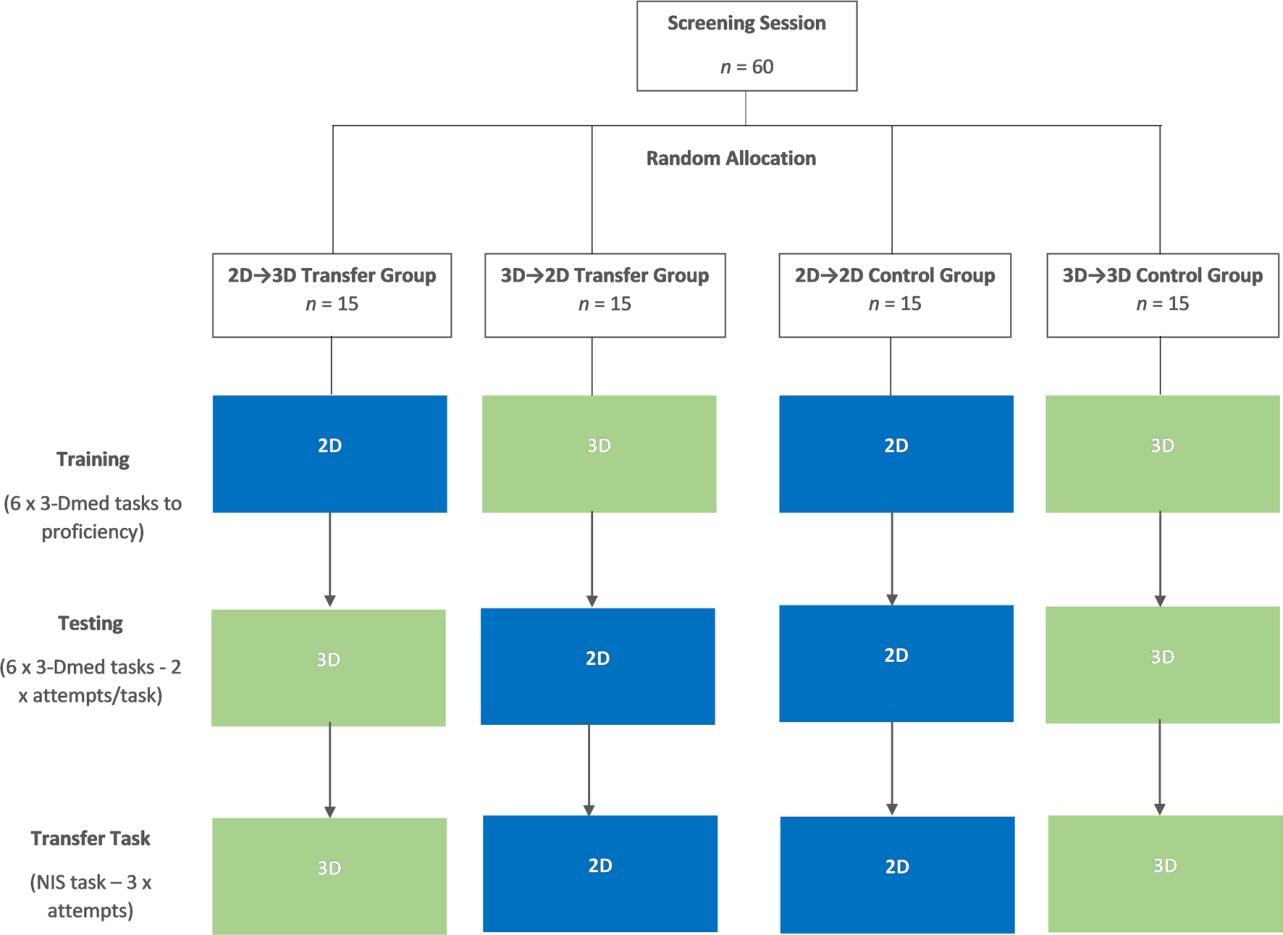
Overview of the study design and procedure showing the viewing mode (2D or 3D) used at each stage of the experiment

Each participant attended a total of four one-on-one sessions with an experimenter. All sessions were conducted in a research lab in a clinical simulation centre at the RBWH, Brisbane, Australia. This included a screening session where background information was obtained and laparoscopically relevant skills and abilities were assessed, followed by two separate training sessions and one testing session (Fig. 1). In the training sessions, the participant completed a series of six laparoscopic training tasks using either a 2D or 3D viewing mode, depending on their experimental group. The six tasks were practised in a set order (i.e. the first three tasks in session one and the last three in session two). Each task was performed repeatedly until the participant was able to complete the task to a pre-defined criterion level of proficiency (on two non-consecutive attempts) before moving on to the next task. During the testing session, the participant performed each of the six tasks twice under test conditions (in the same order as in training), followed by three attempts at a novel task. Participants were also asked to rate their experiences (i.e. perceived workload and physical/visual comfort) following each training and testing session.

### Screening

Participants completed a demographic questionnaire to document their age, gender, hand dominance, use of corrective glasses, occupation, level of medical study, interest in surgery, and their experience in surgery, laparoscopy, suturing, 3D displays, video games, and snooker/billiards. To account for any attrition that may have occurred during the study, a validated measure of psychological grit [22] was also administered. Grit is a characteristic found to positively correlate with residents’ longevity, performance, and well-being during medical training (with higher scores reflecting more grit) [23–25]. Overall, the study did not encounter any such problems with attrition, and grit was not found to be associated with any measure of laparoscopic performance.

Participants’ visual-spatial ability, manual dexterity, stereoacuity, and visual acuity, abilities that have all been associated with novices’ laparoscopic performance [26, 27], were also measured to detect any outliers or significant group-level differences that could potentially confound the results. Visual-spatial ability was assessed using an online adaptation of the Mental Rotations Test (MRT-A) [28]. The MRT-A evaluates participants’ ability to visualise and mentally manipulate a 2D image of a 3D object in space, and is a well-established test of visual-spatial ability that has been utilised in prior laparoscopic research [29–31]. Manual dexterity was measured using the Purdue Pegboard (PP) test (Lafayette Instrument Co). The PP includes four subtests that involve the placement of small pins into holes on a board, and the assembly of pins and washers to assess fine finger dexterity. The reliability and validity of this test has been well established [32, 33]. For both the MRT-A and PP test, higher scores reflect a higher level of ability.

The Randot ® Stereo Test (Stereo Optical, Chicago, IL) was used to assess stereoacuity and ensure that all participant stereopsis was sufficient (between 20 and 200 arc/sec) [34] to effectively use the 3D display. This test presents ten sets of three circles, with each set containing a target circle that appears closer than the others (crossed disparity) when viewed through cross-polarised glasses [18]. The viewer’s task is to identify the target circle. In each successive set, the disparity decreases, and lower scores reflect greater stereoacuity. This test has been found to be a reliable, valid, and sensitive measure of stereoacuity [35, 36].

Participants’ right and left visual acuity were each assessed using the logMAR visual acuity chart (National Vision Research Institute, Melbourne, Australia) at a distance of three metres. The logMAR involves the presentation of a series of five-letter rows, incrementally decreasing in size and spaced in a standardised manner to determine 2D spatial discrimination [37]. A visual acuity score exceeding 9.5 m (or 3/9.5) was considered to be non-normal (as the World Health Organization defines low visual acuity as a logMAR value exceeding 0.5 [38], or a score greater than 9.5 m with a chart placed at a distance of three metres).

### Training

#### Laparoscopic tasks

**Fig. 2:**
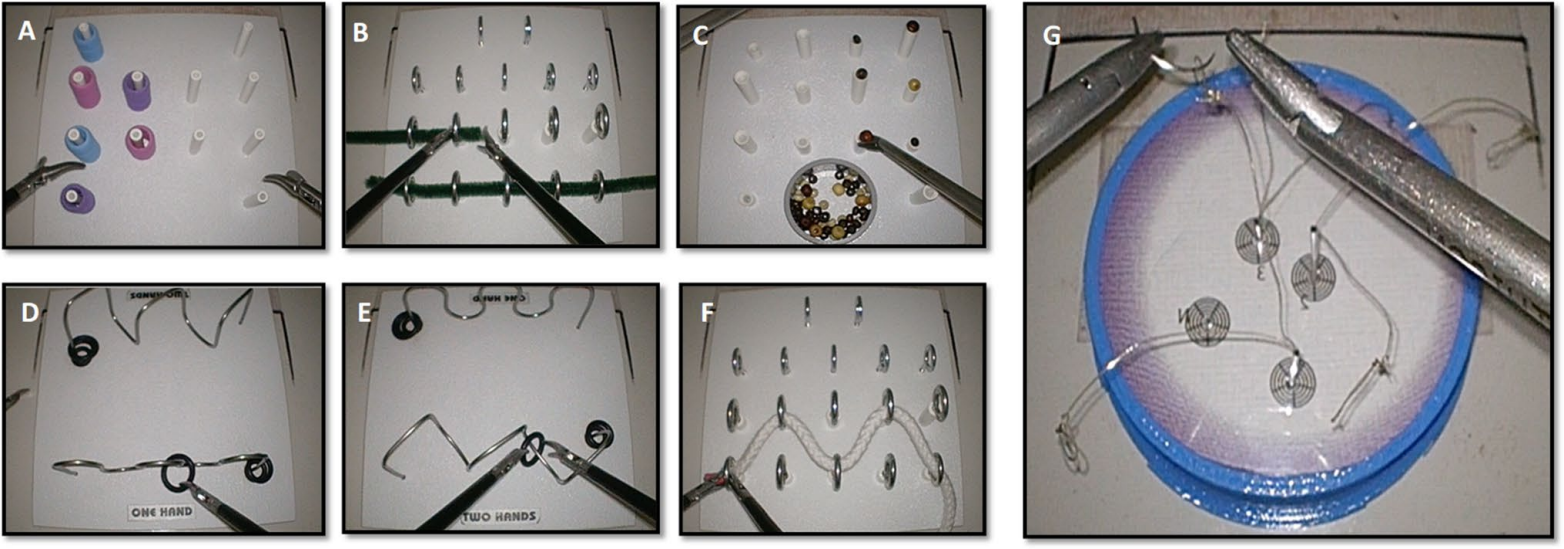
Laparoscopic images of the 3-Dmed tasks used in training and testing **A** Post and Sleeve. **B** Loops and Wire. **C** Pea on a Peg. **D** Wire Chaser (one hand). **E** Wire Chaser (two hands). **F** Zig-zag loop, and the novel task used in testing (**G** Navigating in Space)

Participants performed all six tasks from the 3-Dmed program (3-Dmed®, Franklin, OH, US) with laparoscopic graspers (Fig. 2). The 3-Dmed tasks (i.e. post and sleeve, loops and wire, pea on a peg, wire chaser (one hand), wire chaser (two hands), and zig-zag loop) are intended to train and evaluate skills such as hand–eye coordination, manual dexterity, laparoscopic depth perception, and interactions between the dominant and non-dominant hand through touching, grasping, transferring, placing, navigating, and manoeuvring with laparoscopic graspers [39]. To assess the validity of metrics derived from the use of these 3-Dmed tasks, Schreuder et al. [39] compared the performance scores (i.e. time + error penalties) of groups of laparoscopic experts, intermediates, and novices on the six exercises. They found that the tasks effectively discriminated and reflected the different levels of expertise as the expert group performed significantly better than the novice group on all exercises [39]. Therefore, we employed the average performance scores from this expert group as the criterion level of proficiency in the current study, and all training instructions and error parameters defined by Schreuder et al. [39] were adopted here (see Appendix 1 for task descriptions and criterion proficiency scores).

### Testing

#### Laparoscopic tasks

In addition to repeating the six 3-Dmed tasks twice, participants completed a novel task developed and validated by Sakata et al. [18, 40], known as “Navigating in Space” (NIS). The objective of this task is to measure fine dexterity and instrument control. It was used here to assess whether the training or testing viewing mode impacted novices’ ability to transfer previously acquired skills to a novel task. The task required participants to hold a needle-holder in each hand and pass a curved needle through six 2 mm loops at the tip of a monofilament suture in a pre-defined sequence [18, 40] (Fig. 2). This task was completed a total of three times at the end of the testing session. This included a practice attempt to allow participants to become familiar with the task, followed by two attempts under test conditions. A 10-min time limit was applied to each attempt at this task.

### Outcome measures

#### Repetitions

The total number of repetitions taken to reach the pre-defined level of proficiency across all six tasks was calculated as a measure of training efficiency.

#### Performance scores

The total time taken and the total time penalties incurred for errors across all training repetitions were combined to provide a total performance score for training (in seconds). The total time and the total time penalties incurred across all 12 test attempts (two attempts per 3-Dmed task) were also combined to provide a total performance score for testing (in seconds). Lower scores indicated more efficient and more accurate performances.

#### Novel task performance

Performance on the novel (NIS) task was measured by the total time taken to pass the suture through all six loops, up to a maximum of 600 s (10 min). The times from the two test attempts were then combined to provide a total NIS score (in seconds).

#### Simulator comfort

The Simulator Sickness Questionnaire (SSQ) [41] was administered after each training and testing session, to investigate the impact of 2D and 3D viewing modes on physical/visual comfort. The SSQ was chosen as it is the only validated measure that addresses the symptoms specific to simulation use [41].

#### Workload

With stress and mental workload found to negatively impact laparoscopic task performance [42], the NASA Task Load Index (NASA-TLX) [43] was administered at the end of each training and testing session to measure the self-perceived workload associated with the tasks. The NASA-TLX comprises six rating scales measuring mental demand, physical demand, temporal demand, performance, effort, and frustration. This tool has been extensively validated across a number of industries (including the medical sector) and is now the gold standard for workload assessment [40, 44].

### Equipment and setup

A PC was used to present instructional videos on how to complete the 3-Dmed simulation-based exercises (derived from Schreuder et al. 2011) [39]. All tasks were performed in a box trainer using two laparoscopic graspers (3-Dmed tasks) or needle-holders (NIS task). An Olympus Endoeye Flex 3D Imaging System (Olympus Corporation, Tokyo, Japan) was used to provide the light source, laparoscope, video processors, recording device, and monitor. This system allowed either a 2D or 3D image to be displayed, ensuring consistency in the size and clarity of images shown across conditions. A one metre distance between the display and the participant was continuously maintained to standardise picture clarity and focus across participants. This distance was chosen as it has been found to optimise performance relative to longer distances (e.g. three metres) [18]. Additionally, the height of the monitor was adjusted to ensure that each participant’s natural gaze was perpendicular to the centre of the screen to minimise crosstalk (as per the findings and recommendations of Sakata et al. 2016) [45]. A stopwatch was used to record participants’ time on each task.

### Statistical analysis

Statistical analyses were conducted using SPSS® version 25 (IBM Corp, 2017, Armonk, NY: USA), with α set at 0.05. A chi-square test was used to evaluate categorical data (i.e. gender). A series of one-way ANOVAs was used to identify differences between the four groups on continuous demographic/screening variables, simulator comfort, workload, repetitions, and performance scores during training and testing. One-way ANOVAs were employed to ensure that all appropriate comparisons could be made between training and test performance (e.g. conducting baseline checks for training differences that could have a confounding effect on test results). All significant main effects were followed up with a series of post hoc *t* tests using the Bonferroni–Holm sequential adjustment method [46]. However, the main effect of a demographic variable that violated the ANOVA assumption of equal group variances (i.e. right visual acuity) was followed up using the Games–Howell adjustment method [47]. Effect sizes (*η*^2^) were calculated to further interpret the main effects (*η*^2^ ≥ 0.01, 0.06, and 0.14 reflect small, medium, and large effects, respectively) [48].

Following normality checks and visual examination of the data, one participant was defined as an “extreme” outlier based on Tukey’s (1977) Boxplot rule [49], with scores falling more than three times the length of the interquartile range from the group mean (or equivalent to 4.67 standard deviations) [50]. Given the moderate sample size, and a lack of significant skew in the group’s data to account for this deviation, further investigations were conducted. Subsequent analyses revealed that the participant’s manual dexterity score was more than three standard deviations below the normative mean of their respective age/sex group [51]. Consequently, as this was the only case of non-normal manual dexterity within the sample, the outlier was removed from the substantive analyses to avoid unexplained physical limitations from influencing the results.

## Results

### Participant characteristics

Overall, there were no significant differences between the four groups across any of the demographic variables or individual difference measures (Table 1).

**Table 1:** Participant characteristics

Variable	2D → 3D Transfer (*n* = 15)	3D → 2D Transfer (*n* = 15)	2D → 2D Control (*n* = 15)	3D → 3D Control (*n* = 15)	*F*	*p*
	M (SD)	M (SD)	M (SD)	M (SD)		
Gender (*n* = male:female)^a^	9:6	8:7	7:8	8:7	0.00	1.00
Age (years)	24.73 (3.94)	24.93 (3.11)	24.33 (2.61)	25.13 (3.44)	0.16	0.923
Year of medical study (0–4 years)	1.80 (0.68)	1.73 (0.80)	2.13 (00.74)	1.93 (0.88)	0.77	0.516
Surgical interest (low “1”–high “5”)	3.67 (0.72)	3.87 (0.99)	4.13 (0.99)	3.87 (0.74)	0.72	0.542
No. of surgeries observed	0.73 (0.46)	0.47 (0.52)	0.87 (0.35)	0.67 (0.49)	1.45	0.239
No. of dummy sutures completed	3.07 (2.79)	2.33 (3.11)	5.60 (7.39)	3.47 (5.33)	1.18	0.328
No. of live sutures completed	1.60 (4.14)	0.33 (0.82)	3.60 (12.85)	0.60 (0.99)	0.72	0.546
3D experience (h/last year)	0.93 (1.71)	1.80 (2.34)	2.67 (7.39)	8.60 (25.73)	0.99	0.401
Video-game play (h/last year)	169.27 (244.19)	86.13 (143.73)	88.80 (153.03)	84.47 (112.25)	0.89	0.454
Snooker play (h/last year)	11.33 (22.37)	13.60 (30.25)	33.87 (63.20)	11.07 (21.24)	1.24	0.305
Grit (score from 8 to 40)^b^	28.07 (4.03)	30.73 (3.69)	31.27 (3.73)	28.87 (4.81)	2.06	0.116
Visual-spatial ability (score out of 24)^b^	12.00 (5.35)	12.53 (4.67)	13.27 (5.50)	13.93 (3.97)	0.44	0.722
Dexterity—right (score out of 25)^b^	15.07 (1.67)	15.33 (1.54)	15.47 (1.89)	15.07 (1.94)	0.19	0.901
Dexterity—left (score out of 25)^b^	14.40 (1.68)	14.00 (1.69)	14.60 (1.76)	14.07 (2.28)	0.34	0.795
Dexterity—both (score out of 25)^b^	12.00 (1.73)	12.27 (1.28)	12.07 (1.67)	12.07 (1.83)	0.07	0.974
Dexterity—assembly (score out of 100)^b^	43.00 (5.48)	41.40 (3.85)	43.40 (4.67)	44.00 (9.89)	0.45	0.718
Dexterity – L + R + both (score out of 75)^b^	41.47 (4.27)	41.60 (3.62)	42.13 (4.55)	41.20 (5.53)	0.11	0.953
Stereoacuity (20–400)^c^	37.67 (21.29)	29.67 (13.56)	34.67 (15.86)	31.33 (15.52)	0.67	0.572
Visual Acuity—right (1.5–30)^c^	2.40 (0.46)	2.81 (0.60)	3.51 (1.99)	2.37 (0.48)	3.53	0.021*
Visual acuity—left (1.5–30)^c^	2.47 (0.62)	3.07 (0.87)	3.50 (2.44)	2.40 (0.49)	2.24	0.094

*df* = (3, 56) for all analyses

^a^Chi-square test

^b^Higher score reflects a greater level of the ability

^c^Lower score reflects a greater level of acuity

^*^Games–Howell post hoc tests revealed no significant group differences (all *p*’s > 0.10)

### Training

#### Repetitions

Analyses revealed a significant main effect of group on the total number of repetitions taken to reach proficiency in training (Table 2).

**Table 2:** Training data: mean repetitions, total performance scores, simulator comfort, and perceived workload across the four groups

Measure	2D → 3D transfer	3D → 2D transfer	2D → 2D control	3D → 3D control	*F*	*p*	*η*^2^	Post hoc tests	Comparison	Result	*p*^a^
	M (SD)	M (SD)	M (SD)	M (SD)				Purpose			
Total repetitions	34.07 (11.99)	22.40 (5.60)	35.67 (12.51)	23.40 (8.19)	7.27	** < .001**	.28	*Baseline checks*	2D → 3D vs 2D → 2D 3D → 2D vs 3D → 3D	N/A N/A	1.00 1.00
								*2D vs. 3D training*	2D → 3D vs 3D → 2D 2D → 3D vs 3D → 3D 3D → 2D vs 2D → 2D 2D → 2D vs 3D → 3D	**2D > 3D** **2D > 3D** **2D > 3D** **2D > 3D**	**.008** **.015** **.006** **.006**
Total performance score (s)	4389.67 (2286.89)	2470.53 (730.25)	4670.33 (2083.22)	2522.47 (1360.36)	6.99	** < .001**	.27	*Baseline checks*	2D → 3D vs 2D → 2D 3D → 2D vs 3D → 3D	N/A N/A	1.00 1.00
								*2D vs. 3D training*	2D → 3D vs 3D → 2D 2D → 3D vs 3D → 3D 3D → 2D vs 2D → 2D 2D → 2D vs 3D → 3D	**2D > 3D** **2D > 3D** **2D > 3D** **2D > 3D**	**.006** **.006** **.016** **.016**
Simulator sickness score (1^st^ training)	2.07 (2.12)	1.60 (1.50)	2.47 (2.56)	2.07 (2.19)	.417	.742	.02	N/A	N/A	N/A	N/A
Simulator sickness score (2^nd^ training)	1.93 (1.87)	1.00 (1.25)	1.80 (1.97)	2.47 (2.61)	1.40	.254	.07	N/A	N/A	N/A	N/A
NASA-TLX score (1^st^ training)	26.07 (5.17)	22.53 (4.56)	27.60 (5.12)	24.80 (5.62)	2.61	.060	0.12	N/A	N/A	N/A	N/A
NASA-TLX score (2^nd^ training)	24.13 (4.73)	23.67 (6.40)	25.00 (5.37)	25.60 (5.83)	0.36	0.785	0.02	N/A	N/A	N/A	N/A

Bolded items mean group differences are significant at a level of p < 0.05; and italicised headings reflect the purpose of each group of post-hoc tests

*df* = (3, 56) for all analyses

^a^Adjusted *p* values based on Bonferroni-Holm Sequential Method (*α* = .05)

##### Baseline checks

As expected, post hoc tests indicated that there was no significant difference in the number of repetitions between the two groups trained in 3D (the 3D → 3D and 3D → 2D groups), or between the two groups trained in 2D (the 2D → 2D and 2D → 3D groups) (Table 2).

##### 2D vs. 3D training

Further post hoc tests revealed that the 3D-trained groups (the 3D → 3D and 3D → 2D groups) took significantly fewer repetitions to reach proficiency than the 2D-trained groups (the 2D → 2D and 2D → 3D groups) (Table 2).

#### Total performance score

**Fig. 3:**
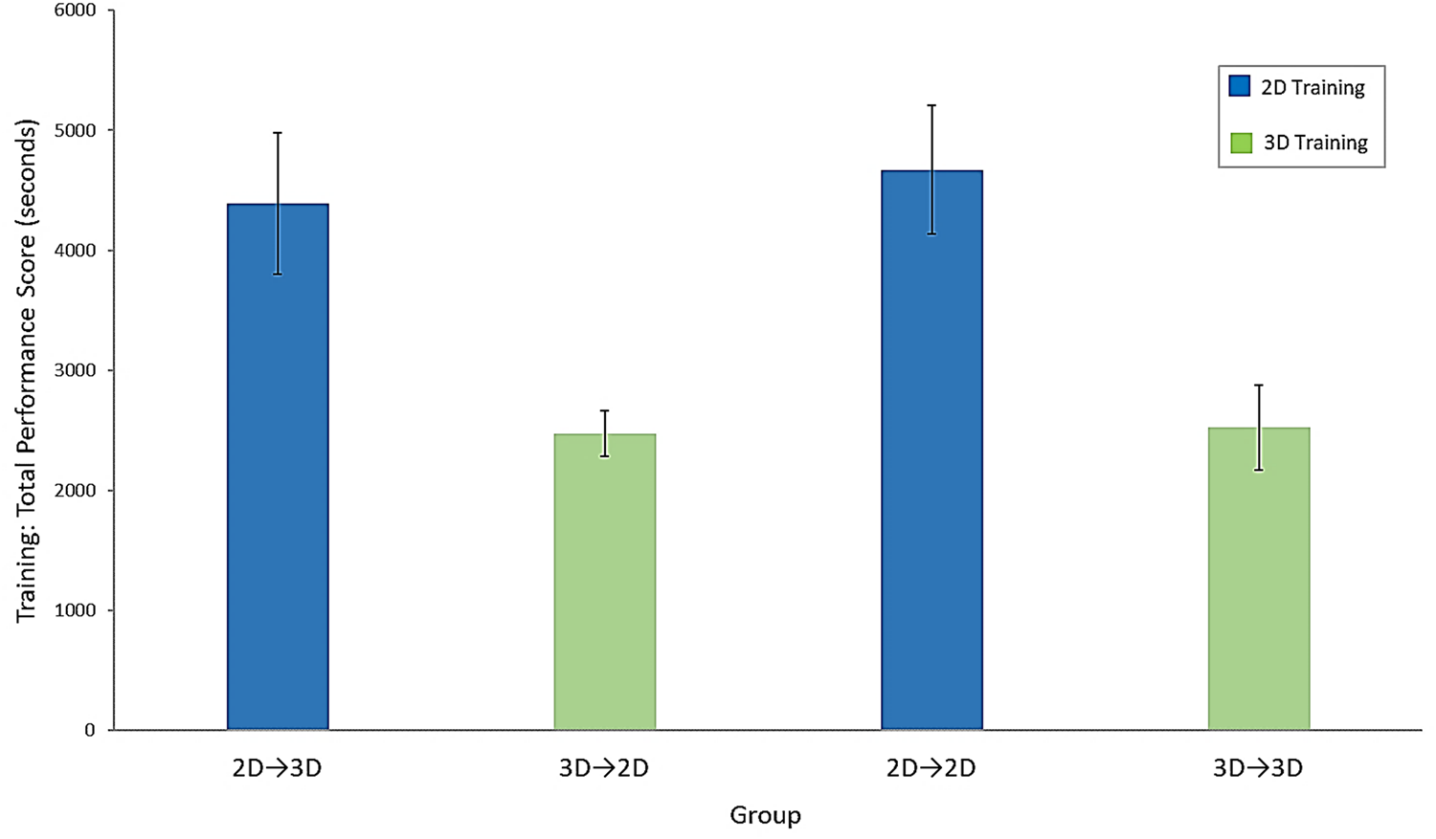
Training data: mean total performance scores across the four groups. Error bars represent standard errors

A significant main effect of group on total performance scores (time to complete tasks + time penalties for errors) in training to proficiency was also revealed (Fig. 3, Table 2).

##### Baseline checks

As expected, post hoc tests indicated that there was no significant difference in total performance scores between the two groups trained in 3D (the 3D → 3D and 3D → 2D groups), or between the two groups trained in 2D (the 2D → 2D and 2D → 3D groups) (Table 2).

##### 2D vs. 3D training

Further post hoc tests revealed that the 3D-trained groups (the 3D → 3D and 3D → 2D groups) performed significantly better during training (i.e. lower total performance scores) than the 2D-trained groups (the 2D → 2D and 2D → 3D groups) (Table 2).

#### Simulator comfort and workload

There was no significant main effect of group on reported simulator sickness or experienced workload following the two training sessions, indicating that these variables could not account for the group differences in performance (Table 2).

### Test

#### Total performance score

**Fig. 4:**
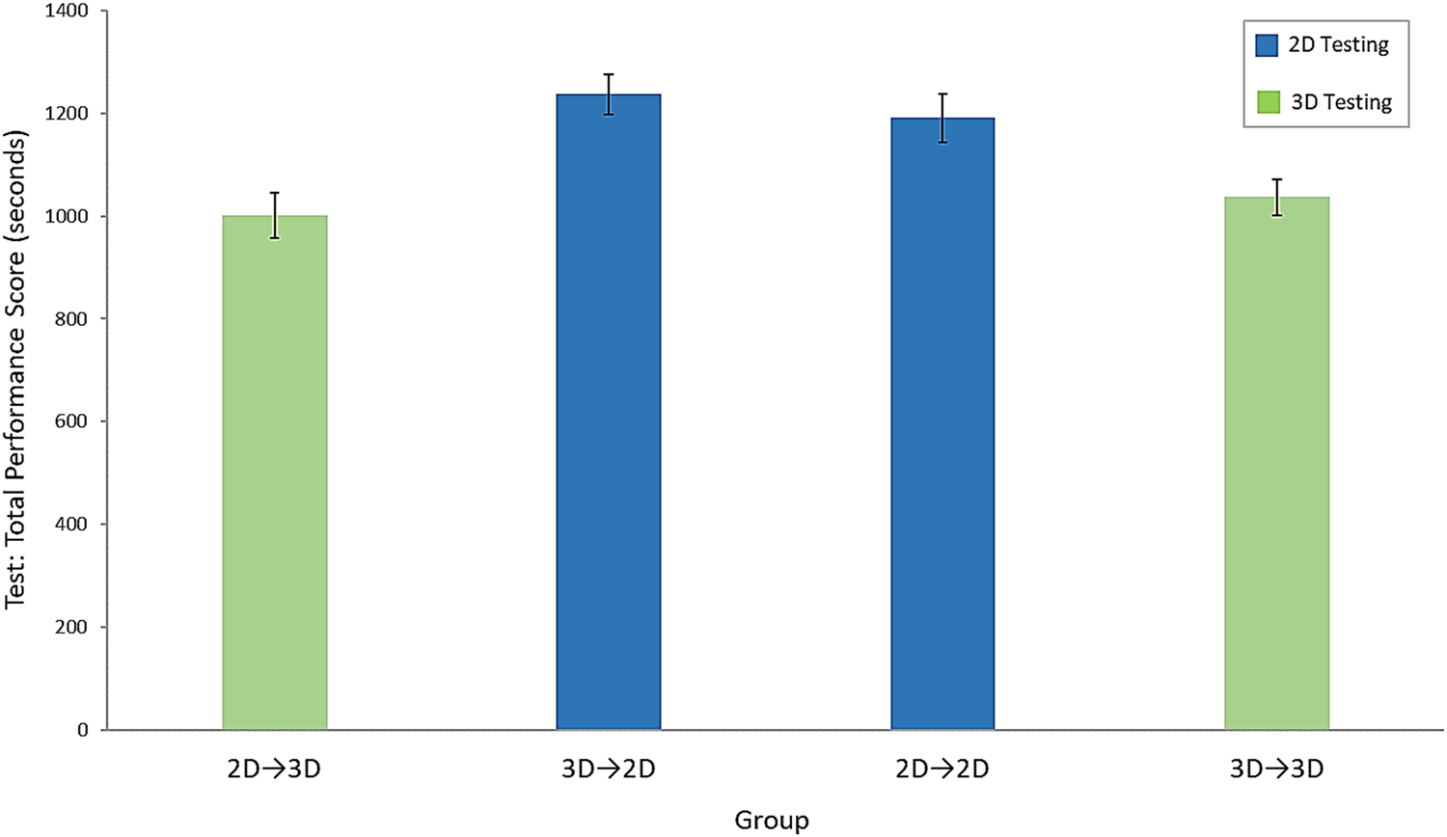
Test data: mean total performance scores across the four groups. Error bars represent standard errors

There was a significant main effect of group on total performance scores (time to complete tasks + time penalties for errors) at test (Fig. 4, Table 3).

**Table 3:** Test data: mean total performance scores, NIS task times, simulator comfort, and perceived workload across the four groups

Measure	2D → 3D transfer	3D → 2D transfer	2D → 2D control	3D → 3D control	*F*	*p*	*η*^2^	Post hoc tests	Comparison	Result	*p*^a^
	M (SD)	M (SD)	M (SD)	M (SD)				Purpose			
Total performance score (s)	1000.80 (168.73)	1237.00 (151.92)	1191.33 (182.51)	1036.67 (137.31)	7.69	** < .001**	0.29	*Control vs. control*	2D → 2D vs 3D → 3D	**2D > 3D**	**0.033**
								*Transfer vs. transfer*	2D → 3D vs 3D → 2D	**2D > 3D**	** < 0.001**
								*Same training, different testing*	2D → 3D vs 2D → 2D 3D → 2D vs 3D → 3D	**2D > 3D** **2D > 3D**	**0.008** **0.005**
								*Different training, same testing*	2D → 3D vs 3D → 3D 3D → 2D vs 2D → 2D	N/A N/A	0.882 0.882
Navigating in space task—total time (s)	368.27 (159.33)	873.73 (170.80)	794.93 (304.75)	405.93 (162.80)	23.44	** < 0.001**	0.56	*Control vs. control*	2D → 2D vs 3D → 3D	**2D > 3D**	** < 0.001**
								*Transfer vs. transfer*	2D → 3D vs 3D → 2D	**2D > 3D**	** < 0.001**
								*Same training, different testing*	2D → 3D vs 2D → 2D 3D → 2D vs 3D → 3D	**2D > 3D** **2D > 3D**	** < 0.001** ** < 0.001**
								*Different training, same testing*	2D → 3D vs 3D → 3D 3D → 2D vs 2D → 2D	N/A N/A	0.623 0.610
Simulator sickness score	2.80 (2.76)	2.33 (2.16)	3.60 (2.64)	2.33 (2.09)	0.91	0.444	0.05	N/A	N/A	N/A	N/A
NASA-TLX score	22.87 (4.03)	27.20 (5.86)	28.33 (4.35)	25.87 (6.94)	2.84	**0.046**	0.13	*Control vs. control*	2D → 2D vs 3D → 3D	N/A	0.654
								*Transfer vs. transfer*	2D → 3D vs 3D → 2D	N/A	0.165
								*Same training, different testing*	2D → 3D vs 2D → 2D 3D → 2D vs 3D → 3D	**2D > 3D** N/A	**0.048** 1.00
								*Different training, same testing*	2D → 3D vs 3D → 3D 3D → 2D vs 2D → 2D	N/A N/A	0.540 1.00

Bolded items mean group differences are significant at a level of p < 0.05; and italicised headings reflect the purpose of each group of post-hoc tests

*df* = (3, 56) for all analyses

^a^Adjusted *p* values based on Bonferroni-Holm Sequential Method (*α* = 0.05)

##### Control vs. control

Post hoc tests revealed a significant difference between the two control groups (3D → 3D and 2D → 2D), with those trained and tested in 3D performing significantly better than those trained and tested in 2D (Table 3).

##### Transfer vs. transfer

Post hoc tests comparing the two transfer groups revealed that the 2D → 3D group performed significantly better during testing than the 3D → 2D group (Table 3).

##### Same training, different testing

Among the groups that trained in 2D, the 2D → 3D transfer group performed significantly better than the 2D → 2D control group during testing. Among those that trained in 3D, the 3D → 2D transfer group performed significantly worse compared to the 3D → 3D control group (Table 3).

##### Different training, same testing

Regardless of the viewing mode used in training, there were no significant differences found between the test scores of the two groups tested in 2D (the 2D → 2D and 3D → 2D groups), and between the two groups tested in 3D (the 3D → 3D and 2D → 3D groups) (Table 3).

#### Novel task

There was a significant main effect of group on the total time taken to complete the novel (NIS) task. Specifically, those tested in 3D (the 3D → 3D and 2D → 3D groups) took significantly less time to complete the novel task than those tested in 2D (the 2D → 2D and 3D → 2D groups). All other NIS comparisons were non-significant (Table 3).

#### Simulator comfort and workload

While there was no significant main effect of group on reported simulator sickness during testing, there was a significant main effect of group on perceived workload (Table 3). The only significant difference was between the 2D → 2D control group and the 2D → 3D transfer group. Specifically, those who transferred from 2D to 3D reported significantly lower perceived workload in testing than those who used 2D only. All other comparisons were non-significant.

## Discussion

Previous studies into the effects of 2D versus 3D viewing modes on laparoscopic skills training were subject to various methodological problems that limit the interpretability of the data. To overcome these limitations, the current study employed control groups to provide greater insights into the effects of viewing mode on laparoscopic performance. In doing so, we confirmed that using a 3D viewing mode did enhance the efficiency of laparoscopic skill acquisition during proficiency-based training compared to 2D. Additionally, the results aligned with the limited research into skill transfer between viewing modes [7, 10–12], with those trained in 2D performing more efficiently and effectively during a post-training test in 3D, compared to those trained in 3D and tested in 2D. Previously, such results have been interpreted as a lack of effective skill transfer from 3D to 2D, and effective or improved skill transfer from 2D to 3D viewing modes [7, 10–12]. However, the omission of control groups in these studies made it impossible to determine whether performance differences in transfer tasks were due to the viewing mode itself, or to the additional practice commonly received in proficiency-based training with 2D. Fortunately, with the inclusion of control groups, we can now rule out the latter interpretation of these results. Specifically, despite receiving the same amount of training in 2D and reaching the same level of proficiency, those who shifted to the 3D viewing mode (2D → 3D group) for testing outperformed those who continued using the 2D viewing mode (2D → 2D group). This suggests that the change in viewing mode significantly impacted performance. More importantly, the current study highlights that, while training to proficiency in 3D may be more efficient than in 2D, it does not necessarily increase or reduce subsequent performance in 2D (i.e. there were no significant differences in performance between the 3D → 2D and 2D → 2D groups in testing). This lack of difference between the respective groups implies that the skills obtained during training are transferred to the alternate mode, but only within the limits of the viewing mode itself. In other words, participants who train in 3D can still interpret and make sufficient use of secondary depth cues when later tested in 2D, as they perform just as well as those trained in 2D. Rather, the data suggest that it is the viewing mode itself that defines performance at any stage.

In order to further broaden the investigation of skill transfer, we also set out to assess whether the skills acquired during training could be transferred to a novel task, and whether the viewing mode impacted this ability. Our results aligned with the findings of Sakata et al. [18], as participants completed the NIS task more efficiently when using the 3D viewing mode compared to 2D, regardless of their training mode. This finding was unsurprising given the beneficial impact of 3D displays in complex tasks/environments and the considerable difficulty of the novel NIS task (with fine precision and perceptual discrimination required to navigate and complete the task successfully) [6, 7, 14, 52]. Furthermore, this may help to explain the one significant pairwise difference in subjective workload found between the groups (i.e. 2D → 3D vs. 2D → 2D) following testing. Specifically, participants who switched to 3D (2D → 3D group) likely found the 3-Dmed tasks easier in testing compared to training (as a result of the additional depth cues), and could subsequently exploit the primary depth cues available to complete the complex novel task with less effort than those who continued using 2D (2D → 2D group). Further, our adherence to the strict methodological recommendations from Sakata et al.’s [19] review paper on 3D viewing (e.g. maintaining optimal viewing positions to avoid potential crosstalk, screening for stereoacuity to ensure participants could successfully use a 3D display) may have prevented any group differences in perceived simulator sickness during training and testing.

Limitations of the study include an inability to re-assess participants following an extended delay to determine whether the viewing mode impacted long-term skill retention. Additionally, while we were able to address the transfer of skills to the alternate viewing mode and compare this to control groups, assessing subsequent performance upon switching back to the original viewing mode may have provided greater insight into how exposure to alternate modes further impacts performance. Finally, given the difficulty of the NIS task, particularly with 2D viewing, it may have proven valuable to use multiple novel tasks with varying levels of difficulty to more thoroughly assess skill generalisability.

The current findings provide additional support for the use of 3D displays to increase the efficiency of proficiency-based training in basic laparoscopic surgical skills. However, unlike prior research, the present data allowed us to confirm that the viewing mode used in training does not significantly impact subsequent performance in an alternate mode. Overall, the current study provides a robust platform for further research into the structure of training to ultimately optimise the early acquisition of laparoscopic skills.

## Electronic supplementary material

Below is the link to the electronic supplementary material.

Supplementary file1 (DOCX 16 kb)
