# Supplementary material for: Laparoscopic skills training: the effects of viewing mode (2D vs. 3D) on skill acquisition and transfer
Source: Surg Endosc. 2020 Sep 2;35(8):4332–44. doi: 10.1007/s00464-020-07923-8 (PMC8263422; doi:10.1007/s00464-020-07923-8)
Supplement: Supplementary file 1 — Supplementary file1 (DOCX 16 kb) [file 464_2020_7923_MOESM1_ESM.docx]

**Appendix 1.** Description, penalties, and criterion proficiency scores of the six 3-Dmed training tasks

| **Task** | **Description** | **Penalties** | **Proficiency Score^a^** |
| --- | --- | --- | --- |
| 1. **Post and sleeve** | Six sleeves are positioned on posts on the opposite side of the board to the participant’s dominant hand. Each sleeve is picked up one-by-one with the non-dominant hand, transferred mid-air to the dominant hand, and placed on a post on the opposite side of the board. Once all six sleeves have been moved to the opposite side, they are picked up with the dominant hand and transferred mid-air to the non-dominant hand, until all sleeves are back on the original side of the board. If a sleeve is dropped on the board, it may be retrieved. If a sleeve is dropped off the board, it may not be retrieved. | - Sleeve dropped *on* the board = 10 second penalty. - Sleeve dropped *off* the board = 20 second penalty. | 120 |
| 1. **Loops and wire** | A pipe cleaner is picked up with the dominant hand and introduced into the first row of loops from the dominant side (e.g., right side if right-handed). Using both hands to manoeuvre, the pipe cleaner is passed through all loops in the first row. The second pipe cleaner is then picked up with the non-dominant hand and introduced into the second row of loops from the non-dominant side. Both hands are again used to pass the pipe cleaner through all loops in the second row. | - Pipe cleaner is passed beside and misses a loop = 10 second penalty. | 86 |
| 1. **Pea on a peg** | Wooden beads are picked up from a cup on the board and placed, one-by-one, onto the 14 pegs. The right side of the board is completed with the right hand and the left side is completed with the left hand. If a bead is dropped on the pegboard, it may be retrieved and placed on a peg. If a bead is dropped off the pegboard, it may not be collected. | - Bead dropped *on* the board = 10 second penalty - Bead dropped *off* the board = 20 second penalty. | 313 |
| 1. **Wire chaser (one hand)** | The dominant hand is used to pick up and move three rings of decreasing diameter, one-by-one, to the other end of a curved wire, starting with the largest ring. | - Ring dropped or lost by the instrument = 10 second penalty. | 69 |
| 1. **Wire chaser (two hands)** | Both the dominant and non-dominant hands are used to pick up and transfer three rings of decreasing diameter, one-by-one, to the other end of a curved wire, starting with the largest ring. Rings are transferred between both hands to manoeuvre around curves in the wire. | - Ring dropped or lost by the instrument = 10 second penalty. | 127 |
| 1. **Zig-zag loop** | A rope is picked up by the dominant hand and introduced into the first row of loops from the dominant side (e.g., right side if right-handed). Using both hands to manoeuvre, the rope is pulled up and passed through the second loop on the second row. The rope is then pulled back down and fed through the third loop on the first row. These actions are continued until the rope has made an “M” shaped or zigzag pattern and has been passed through the last loop on the first row. | - Loop missed = 10 second penalty. | 48 |

Note: ^a^Proficiency scores reflect the mean performance (task completion time + time penalties for errors) of the expert group in Schreuder et al.’s (2011) validation study

**Reference**

- Schreuder, H., van den Berg, C., Hazebroek, E., Verheijen, R., & Schijven, M. (2011). Laparoscopic skills training using inexpensive box trainers: which exercises to choose when constructing a validated training course. *BJOG: An International Journal of Obstetrics & Gynaecology, 118*(13), 1576-1584. doi:10.1111/j.1471-0528.2011.03146.x
